# Emotional speech synchronizes brains across listeners and engages large-scale dynamic brain networks

**DOI:** 10.1016/j.neuroimage.2014.07.063

**Published:** 2014-11-15

**Authors:** Lauri Nummenmaa, Heini Saarimäki, Enrico Glerean, Athanasios Gotsopoulos, Iiro P. Jääskeläinen, Riitta Hari, Mikko Sams

**Affiliations:** a Department of Biomedical Engineering and Computational Science, School of Science, Aalto University, Finland; b Brain Research Unit, O.V. Lounasmaa Laboratory, School of Science, Aalto University, Finland; c Turku PET Centre, Finland; d Advanced Magnetic Imaging Centre, Aalto NeuroImaging, School of Science, Aalto University, Finland

**Keywords:** Emotion, Connectivity, Synchronization, Speech comprehension, Network

## Abstract

Speech provides a powerful means for sharing emotions. Here we implement novel intersubject phase synchronization and whole-brain dynamic connectivity measures to show that networks of brain areas become synchronized across participants who are listening to emotional episodes in spoken narratives. Twenty participants' hemodynamic brain activity was measured with functional magnetic resonance imaging (fMRI) while they listened to 45-s narratives describing unpleasant, neutral, and pleasant events spoken in neutral voice. After scanning, participants listened to the narratives again and rated continuously their feelings of pleasantness–unpleasantness (valence) and of arousal–calmness. Instantaneous intersubject phase synchronization (ISPS) measures were computed to derive both multi-subject voxel-wise similarity measures of hemodynamic activity and inter-area functional dynamic connectivity (seed-based phase synchronization, SBPS). Valence and arousal time series were subsequently used to predict the ISPS and SBPS time series. High arousal was associated with increased ISPS in the auditory cortices and in Broca's area, and negative valence was associated with enhanced ISPS in the thalamus, anterior cingulate, lateral prefrontal, and orbitofrontal cortices. Negative valence affected functional connectivity of fronto-parietal, limbic (insula, cingulum) and fronto-opercular circuitries, and positive arousal affected the connectivity of the striatum, amygdala, thalamus, cerebellum, and dorsal frontal cortex. Positive valence and negative arousal had markedly smaller effects. We propose that high arousal synchronizes the listeners' sound-processing and speech-comprehension networks, whereas negative valence synchronizes circuitries supporting emotional and self-referential processing.

## Highlights

• We model how emotional speech synchronizes brains across listeners.
• Participants listened to emotional and neutral narratives during fMRI scan.
• Arousal synchronized auditory cortices and Broca's area.
• Valence synchronized limbic system, prefrontal, and orbitofrontal cortices.
• Valence and arousal triggered distinct patterns of dynamic functional connectivity.

## Introduction

Storytelling provides humans a powerful means for sharing emotions with others. When a grown-up daughter reveals over the dinner table that she will be marrying the man of her dreams soon, excitement and joy sweep promptly across the family members, whereas the news of the sudden death of a beloved colleague and friend instantly turns the cheerful day grey and depressing. According to prevailing theoretical views, evolutionarily old brain circuits govern affective responses to biologically determined survival-salient events, such as encounters with predators or possible mating partners (Damasio, 1994, Damasio, 1998, Tsuchiya and Adolphs, 2007). These circuitries nevertheless interact with higher-order cognitive and linguistic processing, and thus listening to others' descriptions of emotional events may trigger vivid emotional imagery of the described episodes. Here the emotional significance associated with certain episodes likely activates the brain's emotion circuits (Costa et al., 2010, Jabbi et al., 2008, Wallentin et al., 2011) and associated changes take place in the sensorimotor and visceral systems (Vrana and Lang, 1990), enabling us to ‘catch’ the emotions described in spoken and written language.

### Catching emotions from spoken narratives

We do not currently understand the neural basis underlying the high degree of similarity in thoughts and feelings of subjects listening to others' emotional narratives. Neuroimaging studies using nociceptive (Jackson et al., 2005, Saarela et al., 2007, Singer et al., 2004) and chemosensory (Jabbi et al., 2007, Wicker et al., 2003) stimulation have revealed activation of overlapping brain areas both when the subjects experience emotions and when they observe others experiencing the corresponding emotions. It has remained, however, untested whether sharing emotional states induced by speech provides the observers a somatovisceral framework (Hatfield et al., 1994, Keysers et al., 2010, Niedenthal, 2007) that facilitates understanding the intentions and actions (i.e., ‘tuning in’ or ‘syncing’) with other individuals via contextual understanding. This hypothesis is supported by work showing how different individuals' brain activity in the limbic emotion circuits, as well as in the early visual and dorsal attention networks, becomes synchronous when the individuals view biologically significant emotional events in movies (Nummenmaa et al., 2012). Tentatively, such emotion-driven synchronization of brain activation could facilitate humans to take the mental and sensorimotor perspectives of others and even predict their actions (Hasson et al., 2012, Nummenmaa et al., 2012).

It nevertheless remains unresolved which of these effects reflect emotional processing in specific sensory domains, and which in turn reflect modality-independent synchronization effects triggered by affective stimulation. It is indeed likely that the prior observations on high arousal synchronizing brain's visual and dorsal attention circuits (Nummenmaa et al., 2012) would reflect emotional visual stimuli that capture visual attention similarly in all individuals (Nummenmaa et al., 2006). Similarly, listening to emotional narratives could be expected to modulate intersubject synchronization of the brain's auditory cortices and language circuits, because auditory emotional cues also capture attention in an automatic fashion and consequently amplify sensory processing of the sounds (Thierry and Roberts, 2007). On the contrary, enhanced intersubject synchrony in the cortical midline systems (medial prefrontal cortex; mPFC and precuneus) could reflect modality-independent similarity of brain activation brought about by emotions. These regions are consistently activated by emotional stimulation in different sensory modalities, likely contributing to the subjective experience of emotion (see review in Kober et al., 2008). Furthermore, together with the posterior cingulate cortex (PCC), the mPFC and precuneus form the medial part of the ‘default mode’ network, which is linked to self-referential processing in more general sense (Buckner and Carroll, 2007, Northoff and Bermpohl, 2004).

### Brain networks encoding valence and arousal

Functional neuroimaging studies have revealed that distinct cerebral networks underlie the valence and arousal dimensions of human emotions. In general, increased arousal seems to be associated with enhanced responses in the sensory cortices, amygdala and thalamus, whereas valence is associated with enhanced frontal and orbitofrontal responses (Anderson et al., 2003, Viinikainen et al., 2012, Wallentin et al., 2011). Yet the large-scale functional networks supporting emotional processing have remained largely unknown. Prior studies have typically constrained the analysis to a limited number of ROIs due to relatively low power of the conventional boxcar and event-related designs in connectivity analyses (e.g. Eryilmaz et al., 2011, Passamonti et al., 2008, Tettamanti et al., 2012). However, studying functional connectivity changes unfolding during prolonged naturalistic emotional stimulation such as spoken narrative provides an elegant solution to this problem. Spoken narratives elicit strong, time-variable emotional reactions, whose time series can be accurately tracked (Wallentin et al., 2011). Because our recently introduced dynamic connectivity analysis techniques can take into account the entire time-dependent response profile rather than the mere magnitude of specific response modulation (Glerean et al., 2012), they can be used for modelling the effects of the aforementioned changes in emotion states on large-scale, dynamic connectivity changes. Specifically, it could be predicted that during emotionally arousing episodes of the narratives, the connectivity between the brain's language circuits as well as the limbic emotion systems would increase, reflecting enhanced sensory gain and more detailed semantic processing of the incoming narrative. Similarly, connectivity between the language and sensorimotor and visceral systems during emotionally intense episodes could enable triggering the physiological affective response (Vrana and Lang, 1990) and the corresponding bodily sensations of emotions in the observer (Nummenmaa et al., 2014a).

**Fig. 1:**
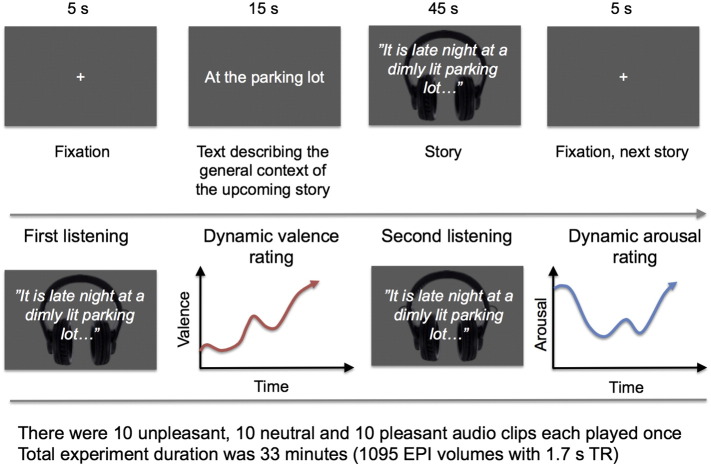
Experimental design for fMRI (top) and subjective emotional ratings (bottom). Participants listened to short spoken narratives describing pleasant, unpleasant and neutral events. The discourses were preceded by a 5-s presentation of a fixation cross and by a 15-s presentation of a text that described the general context of the upcoming discourse without revealing details of its actual events. After fMRI, the participants listened to the discourses again, and rated their moment-to-moment experiences of valence (pleasantness–unpleasantness) and arousal.

Here, we used inter-subject phase synchronization (ISPS) and seed-based phase synchronization (SBPS) analysis (Glerean et al., 2012) of functional magnetic resonance imaging (fMRI) data to estimate intersubject synchronization of brain activity and changes in dynamic connectivity of brain areas when subjects were listening to narratives describing emotional events and controlled for acoustic and prosodic confounds (Fig. 1). Rather than studying how emotions flow from one brain to another (e.g. Anders et al., 2011), we focused on the tendency of emotional brain responses to become synchronized across the members of a group which is exposed to similar emotional events (Nummenmaa et al., 2012). We tested two specific hypotheses: 1) When participants listen to semantically affective narratives, synchrony increases in their language- and emotion-related brain circuitries. 2) Dynamic large-scale connectivity of brain networks supporting emotion, language and self-referential processing increases during emotional events in the stories, thus revealing functional networks that change dynamically during emotional processing of narratives.

## Materials and methods

### Participants

The Aalto University Ethics Review Board approved the study protocol, and the study was conducted in accordance with the Declaration of Helsinki. Twenty healthy adults (8 males, ages 19–30, mean age = 25 years, *SD* = 3.4) with normal hearing volunteered for the study. Individuals with a history of neurological or psychiatric disease or current medication affecting the central nervous system were excluded. All subjects were compensated for their time and travel costs, and they signed ethics-committee-approved, informed consent forms.

### Stimuli and experimental design

Fig. 1 describes the stimuli and study design. Thirty 45-s spoken narratives describing unpleasant, neutral and pleasant events, with 10 stories belonging to each emotion category, were chosen from the ANET database (Bradley and Lang, 2007). The original 15-s stories were translated to Finnish and expanded to 45 s each to permit time-variable ISPS and SBPS analyses during the story (see below).

The recorded narratives were read by a neutral female voice that provided no prosodic cues for the affective significance of the story contents. The background noise in the recording room was recorded and equalized with Apple Logic Pro 9 (Apple Inc.), and subsequently, gate and compressor were used to attenuate the background noise during moments of silence and slightly compress the voice dynamic to limit the variance of the sound power. The loudness of each story was normalized according to the peak value. Time series of the stories' low-level acoustic features (loudness and pitch) were assessed with the MIR toolbox (Lartillot and Toiviainen, 2007).

The participants listened to the narratives once while being scanned with fMRI. The order was kept fixed across subjects, as required by the ISPS analyses (see below). Subjects were instructed to listen to the narratives similarly as if they would listen to a radio or podcast, and to try to get involved in the stories by imagining the described events vividly. Each narrative was preceded, for 5 s, by a fixation cross and, for 15 s, by a short text that explained the general setting of the forthcoming narrative without revealing its actual content. The latter epoch served also as a washout period for the emotion elicited by the previous narrative. During fMRI, the stimuli were delivered using Presentation software (Neurobehavioral Systems Inc., Albany, California, USA). The narratives were played to the subjects with an UNIDES ADU2a audio system (Unides Design, Helsinki, Finland) via plastic tubes through porous EAR-tip (Etymotic Research, ER3, IL, USA) earplugs. Sound was adjusted for each subject to be loud enough to be heard over the scanner noise.

### Psychophysiological recordings

Heart rate and respiration were successfully recorded during the fMRI experiment from 12 participants using the BIOPAC MP150 Data Acquisition System (BIOPAC System, Inc.). Heart rate was measured using BIOPAC TSD200 pulse plethysmogram transducer, which records the blood volume pulse waveform optically. The pulse transducer was placed on the palmar surface of the participant's left index finger. Respiratory movements were measured using BIOPAC TSD201 respiratory-effort transducer attached to an elastic respiratory belt, which was placed around each participant's chest to measure changes in thoracic expansion and contraction during breathing. Both signals were sampled simultaneously at 1 kHz using RSP100C and PPG100C amplifiers for respiration and heart rate, respectively, and BIOPAC AcqKnowledge software (version 4.1.1). Respiration and heart signals were then down-sampled to 10 Hz to extract just the time-varying heart and respiration rates (cycles per minute) with the DRIFTER toolbox (Sarkka et al., 2012) after the effects of movement (i.e., spiky signals) were smoothed, when necessary, by interpolating. Finally, mean heart and breathing rates were computed and down-sampled to 5 Hz to match the valence and arousal self-report time series (see below), and correlations were computed between valence, arousal, and heart and breathing rates.

### Behavioural measurements and analysis

After the fMRI experiment, the participants listened to the same narratives again from earphones on a desktop computer and rated their emotional feelings on-line (Fig. 1, bottom). Ratings were not conducted during scanning, because a reporting task is known to influence neural response to emotional stimulation (Hutcherson et al., 2005, Lieberman et al., 2007); yet repeating a specific emotional stimulus has only a negligible effect on self-reported emotional feelings (Hutcherson et al., 2005). Valence and arousal were rated on separate runs. While listening to each narrative, participants used a mouse to move a small cursor at the edge of the screen up and down in order to indicate their current experience; data were collected at 5 Hz. The actual valence–arousal scale was arbitrary, but for the analyses the responses were rescaled to range from one (negative valence/low arousal) to nine (positive valence/high arousal).

### fMRI acquisition and analysis

MR imaging was performed with Siemens MAGNETOM Skyra 3-tesla MRI scanner at the Advanced Magnetic Imaging Centre (Aalto NeuroImaging, Aalto University). Whole-brain data were acquired with T2*-weighted echo-planar imaging (EPI), sensitive to blood-oxygen-level-dependent (BOLD) signal contrast with the following parameters: 33 axial slices, 4-mm slice thickness, TR = 1700 ms, TE = 24 ms, flip angle = 70 deg, FOV = 200 mm, voxel size 3 × 3 × 4 mm^3^, ascending interleaved acquisition with no gaps between slices). A total of 1095 volumes were acquired, preceded by 4 dummy volumes to allow for equilibration effects. T1-weighted structural images were acquired at a resolution of 1 × 1 × 1 mm^3^. Data were preprocessed using FSL software. The EPI images were sinc-interpolated in time to correct for slice time differences and realigned to the first scan by rigid body transformations to correct for head movements. EPI and structural images were co-registered and normalized to the T1 standard template in MNI space (Evans et al., 1994) using linear and non-linear transformations, and smoothed with a Gaussian kernel of FWHM 8-mm.

Because head motion can be a significant confounding factor when considering group comparisons of e.g. functional connectivity (Yan et al., 2013), we computed framewise displacement (FD) index to assess severity of motion artefacts (Power et al., 2012). The FD (M = 0.09 mm, SD = 0.04 mm) values suggested only minimal motion throughout the experiment. Since we used time-varying measures of intersubject similarity and functional connectivity, we also tested whether the components of the FD signal (i.e. the derivates of the motion parameters) were correlated across subjects or whether they were correlated with experimental regressors. Non-parametric permutation tests yielded no significant synchronization of motion across the subject or correlation between motion parameters and regressors, thus essentially ruling out motion confounds. Consequently, we applied the conventional approach for motion correction and simply regressed the six individual motion parameters out from the BOLD time series.

### Inter-subject phase synchronization

Inter-subject phase synchronization (ISPS) was computed using fMRI Phase Synchronization toolbox (Glerean et al., 2012). ISPS is a measure similar to moment-to-moment intersubject correlation (ISC) computed with a sliding temporal window (Kauppi et al., 2010, Nummenmaa et al., 2012) but it has significantly higher temporal resolution (1 TR of fMRI acquisition which is also the theoretically maximum resolution). Therefore, it can be used to estimate instantaneous synchronization of brain activity across individuals. We have previously validated the ISPS technique against conventional ISC analysis (Glerean et al., 2012), and demonstrated recently that in the context of cognitive neuroimaging experiments using boxcar designs similar as in here, the ISPS analysis gives spatially similar results as ISC. However, the ISPS has a significant sensitivity advantage over ISC and can thus reveal synchronization effects not discernible in the ISC analysis (Nummenmaa et al., 2014b).

In the ISPS analysis pipeline the motion-regressed data are first band-pass filtered through 0.04–0.07 Hz to remove noise and because the concept of phase synchronization is meaningful only for narrowband signals. Furthermore, this frequency band overlaps with the ‘slow four’ frequency band which is least affected by physiological noise in the connectivity analysis (Glerean et al., 2012), and analysing functional connectivity with slower frequencies is problematic due to high noise levels. After Hilbert transform, ISPS time series is calculated for each voxel and EPI image in the time series. The voxelwise ISPS time series may be modelled with experimental regressors to estimate the effect of experimental manipulation on regional phase synchronization. As ISPS measures intersubject similarity in phase rather than in statistical terms, such as covariance or correlation (*c.f.* ISC), it is temporally more accurate than sliding-window ISC and also better suited for quantifying intersubject synchronization in blocked designs, where sliding-window ISC would smear signals coming from different blocks. Importantly, phase difference information between voxel pairs can be further used for estimating dynamic functional connectivity (see below).

Time series of valence and arousal were down-sampled to 1 TR and convolved with a gamma function (θ = 1, k = 6) to account for the hemodynamic lag. A simple gamma function rather than a conventional double-gamma HRF was used, because ISPS reflects intersubject similarity rather than the amplitude of hemodynamic activation. ISPS signal thus can have only a positive stimulus-driven deflection, indicating increased similarity, without the undershoot of BOLD signal, and consequently a single gamma function will serve as an effective filter (convolution function) for increasing SNR in the analysis as well as for compensating for the hemodynamic delay. The valence and arousal regressors were then used to predict voxel-wise ISPS time courses in the general linear model (GLM). Resulting regression coefficients were stored in synchronization maps, where voxel intensities reflect the degree to which ISPS is dependent on valence and arousal.

### Representational similarity analysis (RSA) of emotional feelings and ISPS

As participants gave individual valence and arousal ratings for the narratives, we could also directly test whether similarity in brain activation would be associated with similarity in mental (here emotional) states. We compared the representations of the voxel-wise BOLD time series and valence and arousal time series using representational similarity analysis (Kriegeskorte et al., 2008). We computed pairwise similarity matrices of the BOLD time series across subjects for each voxel, as well as arousal and valence time series. We then used RSA to compare the agreement of valence and arousal time series with the voxelwise agreement of BOLD time series, and generated RSA maps where the voxel intensities reflect the degree to which the similarity in the subjective valence or arousal ratings predict the similarity in BOLD time series.

### Seed-based phase synchronization analysis of large-scale functional connectivity

To assess whether valence and arousal are associated with changes of functional connectivity in large-scale brain networks, we estimated dynamic functional connectivity of regional neural time courses using seed-based phase synchronization (SBPS). Significant changes in SBPS do not in themselves indicate the direction or neurochemistry of causal influences between brain regions, nor whether the connectivity is mediated by mono- or poly-synaptic connections, nor structural changes from epoch to epoch. However, they do indicate functional interactions between regional systems, which may or may not be supported by existing anatomical connections. Because calculation of all possible voxelwise connections (~ 3.5 ∗ 10^8^) for each of the 845 time points would be computationally prohibitive at native EPI data resolution, we spatially down-sampled the data to isotropic 6 × 6 × 6-mm^3^ voxels prior to estimating the time-variable functional connectivity. Voxels outside the grey matter were masked out using mean grey matter image obtained from segmented T1 images, thus resulting in a connectivity matrix of 5183 × 5183 voxels.

To reveal those pairs of regions for which the dynamic connectivity depended most strongly on valence and arousal, we computed instantaneous seed-based phase synchronization (Glerean et al., 2012) as a time-varying group measure of connectivity between every pair of voxels in the brain, resulting in a time-variable network of 13429153 connection time series. The aforementioned gamma-convolved valence and arousal regressors were used to predict each connection's time series in the general linear model (GLM) to assess the positive and negative effects of valence and arousal on functional connectivity. The mean voxel-wise connectivity changes were stored in connectivity maps, where link intensities reflect the degree to which SBPS is dependent on valence and arousal. Furthermore, we estimated the amount of overlap between the four aforementioned networks (valence-positive, valence-negative, arousal-positive, arousal-negative) by computing Jaccard pairwise distance between the connectivity matrices, that is, the number of links common in two conditions divided by the total possible number of links in the network.

Statistically significant functional connections were plotted on cortical flatmaps using the Gephi software (Bastian et al., 2009). Voxel-wise average node degrees were stored into a node degree or ‘hub’ maps, where voxel intensities reflect how many connections from each voxel were statistically significantly modulated by valence and arousal. Whole-brain connectivity maps were also summarised as connectivity matrices by grouping nodes into predefined anatomical regions (using AAL atlas) and considering only the strongest 20% of hubs that were defined using degree centrality and betweenness centrality at connection density of 10% (Rubinov and Sporns, 2010).

Statistical significance of the association between emotion ratings and ISPS and SBPS time series was based on a fully nonparametric voxel-wise permutation test for *r* statistic (Kauppi et al., 2010). We approximated the full permutation distribution independently for each voxel, for ISPS, and independently for each connection, for SBPS, with 10,000 permutations per voxel (or connection) using circular block resampling (Politis and Romano, 1992). For ISPS we corrected the resulting *p*-values using Benjamini–Hochberg False Discovery Rate (FDR) procedure based multiple comparisons correction with independence (or positive dependence) assumption. Due to the large number of links, we used positive FDR (Storey and Tibshirani, 2003) of (q < 10%) to control false discovery rate for the connectivity time-series; this choice is equivalent to the cluster network correction, which takes into account the large number of links in the network without being overly conservative (Zalesky et al., 2010). For validation purposes, we also computed the cluster-based thresholding using permutation testing; this approach gives actually more relaxed threshold than conventionally used q < 0.1, so that the density of the network makes it difficult to extract the desired network properties from the data. Because the number of links is large (~ 13.4 ∗ 10^6^ connections per network), cluster-based thresholding is computationally prohibitive and not practical for routine use, particularly as thresholding at q < 0.1 gives a good approximation of the ‘true’ permutation-based threshold which does not yet make the networks too sparse for computing graph-theoretical measures.

### BOLD responses evoked by valence and arousal

To validate that the emotional narratives triggered reliable activity in the brain's emotion-related circuitry, we modelled the effects of emotional valence and arousal in GLM using SPM8 software. A random-effects model was implemented using a two-stage process (first and second levels). For each participant, we used the GLM to assess regional effects of the valence and arousal parameters on BOLD indices of activation. The model included the orthogonalized valence and arousal time series and effects of no interest (realignment parameters) to account for motion-related variance. To keep the analysis consistent with the ISPS by valence and arousal analysis, time series averaged across subjects were used as predictors, given that individual time series could not be used to predict the ISPS time series of brain activity. Low-frequency signal drift was removed using a high-pass filter (cutoff 256 s), and AR(1) modelling of temporal autocorrelations was applied. Individual contrast images were generated for the positive and negative effects of valence and arousal. The second-level analysis used these contrast images in a new GLM and generated statistical images, that is, SPM-t maps. With balanced designs at first level (i.e. similar events for each subject, in similar numbers), this second-level analysis closely approximates a true mixed-effects design, with both within- and between-subject variance. Statistical threshold was set at T > 3.0 and *p* < 0.05, FDR corrected at cluster level.

## Results

### Self-reports and psychophysiology

**Fig. 2:**
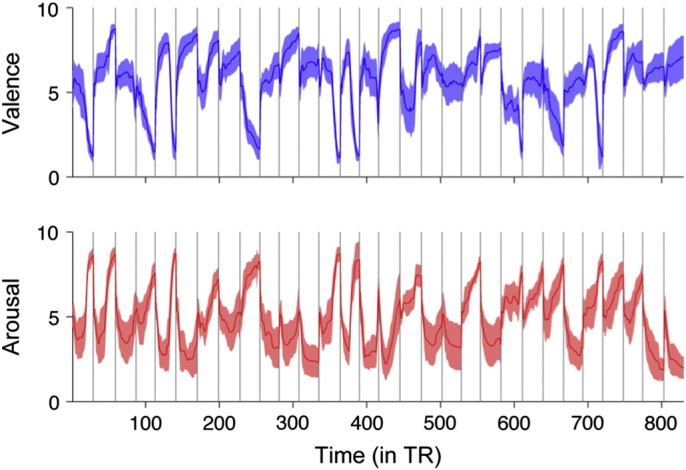
Means and 95% confidence intervals for valence and arousal rating (1–9) while the subjects were listening to the narratives. Vertical lines denote breaks between stories. Note that time is given as multiples of TR (1 TR = 1.7 s).

Behavioural ratings (Fig. 2) confirmed that listening to the narratives elicited strong emotions that varied over time. Mean valence ranged from 1.14 to 8.80, and mean arousal from 1.83 to 8.72. As usual (Lang, 1995), valence and arousal were negatively correlated (*r* = − 0.51, *p* < 0.001). Analysis of the heart and respiration rate time series revealed that emotional sensations were associated with reliable psychophysiological response patterns: valence correlated negatively with heart rate (*r* = − 0.24, *p* < 0.05) and respiration rate (*r* = − 0.32, *p* < 0.05), whereas arousal correlated positively only with respiration (*r* = 0.32, *p* < 0.05) but not with heart rate (*p* > 0.05). Importantly, low-level acoustic properties of the narratives (pitch and loudness) were not associated with valence (*r*_pitch_ = − 0.03, *r*_loudness_ = − 0.002, *p*s > .05) or arousal (*r*_pitch_ = − 0.02, *r*_loudness_ = − 0.04, *ps* > .05). Because BOLD signal was filtered before running the phase synchrony analyses, we also verified that the valence and arousal time series convey information at similar frequency bands as does the ISPS. This analysis (Supplementary Fig. 1a) revealed that the emotions indeed fluctuate at a rate that is included in the bandpass filtered ISPS data, thus confirming that the corresponding analyses are conceptually meaningful.

### Emotion-dependent instantaneous intersubject phase synchronization

**Fig. 3:**
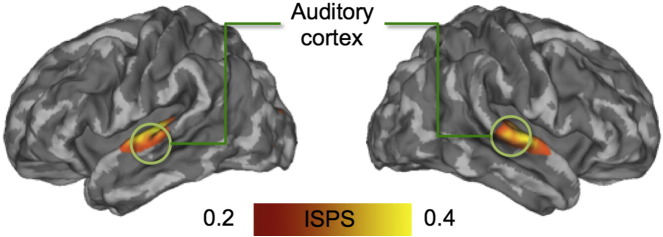
Brain regions showing statistically significant (*p* < 0.05, FDR corrected) group-level ISPSs during listening of the narratives.

**Fig. 4:**
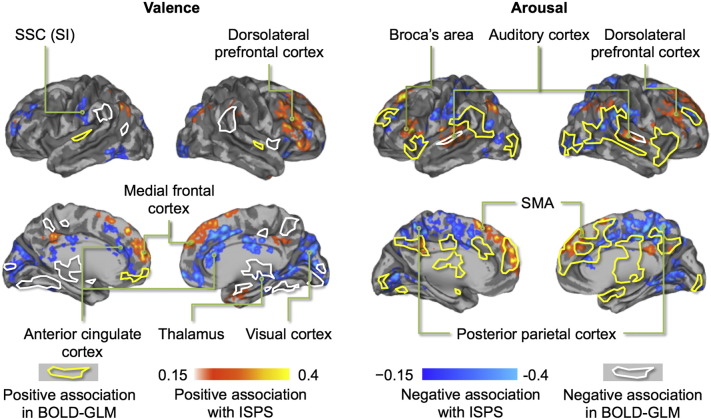
Brain regions where ISPS was statistically significantly associated with self-reported valence and arousal time series during listening to the stories (*p* < 0.05, FDR corrected). Hot colours show positive and cool colours negative associations. The yellow (positive) and white (negative) outlines show the respective associations in conventional BOLD-GLM. Note: SMA = supplementary motor area, and SSC = somatosensory cortex.

When mean ISC was computed for the narrative part of the experiment, subjects' brain activity was time-locked only in the auditory cortices, with synchronization being markedly absent elsewhere in the brain (Fig. 3). Next, we tested whether the degree of ISPS would be associated with the participants' emotional state. This analysis^1^ revealed a regionally-selective association between emotional valence and ISPS, and emotional arousal and ISPS. As valence decreased from positive to negative, ISPS increased in regions involved in emotional processing (thalamus, anterior cingulate cortex, primary somatosensory cortex (SI)) as well as in midline regions implicated in self-referential processing (Fig. 4, left panel and Table S-1). When valence increased from negative to positive, ISPS increased in dorsolateral and medial prefrontal cortices. The level of emotional arousal was positively associated with ISPS in medial prefrontal cortex and in regions involved in auditory and language processing (auditory cortex, Broca's area; Fig. 4 right panel and Table S-1). When arousal decreased from positive to negative, ISPS increased in associative visual cortices, middle cingulum, precuneus and supplementary motor cortices.

### Representational similarity analysis of emotional feelings and ISPS

The RSA analysis revealed that valence and arousal time series showed matching patterns of similarity: when participants were similar with respect to their valence ratings, they were also similar with respect to their arousal ratings. Accordingly, RSA with ISPS revealed highly concordant results for valence and arousal. Enhanced similarity in emotional feelings of both valence and arousal was associated with enhanced BOLD similarity in visual (lingual and calcarine gyri) as well as bilateral auditory (superior temporal and Heschl's gyri) cortices, and precuneus and middle cingulate cortex. Furthermore, enhanced similarity in emotional valence resulted in enhanced BOLD similarity in the supplementary motor area.

### Emotion-driven changes in seed-based phase synchronization connectivity

**Fig. 5:**
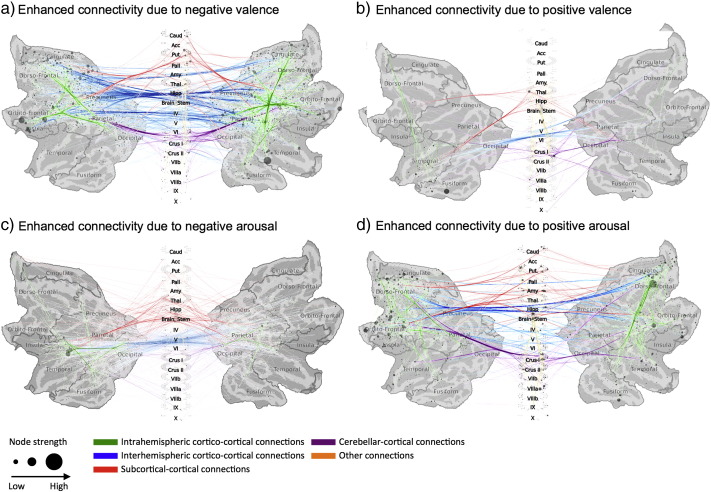
Connectivity graphs showing brain regions whose functional interconnectivity increased as a function of negative (left) or positive (right) emotional valence (top row, a–b) and arousal (bottom row, c–d). Colour coding denotes link type, and node circle size indicates node strength. The data are thresholded at q < 0.1, FDR corrected.

**Fig. 6:**
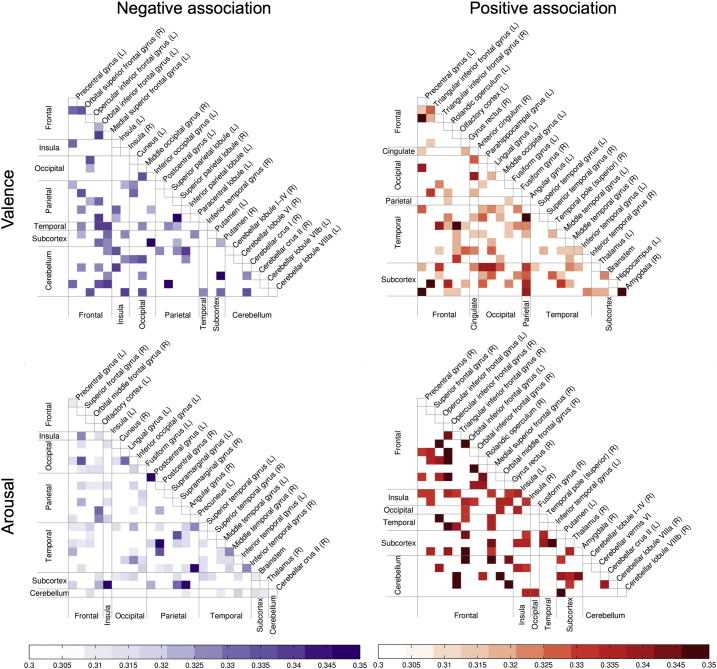
Reduced connectivity graphs showing the main network hubs whose interconnectivity increased as a function of negative (left) or positive (right) emotional valence (top row, a–b) and arousal (bottom row, c–d).

**Fig. 7:**
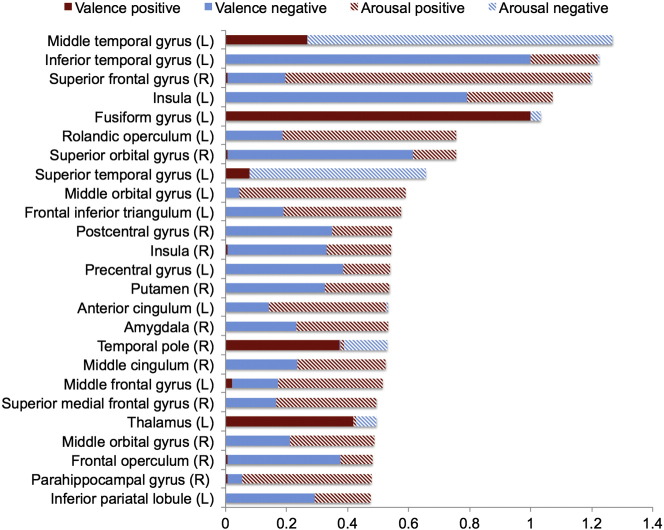
Normalized node degree of the largest hubs of the positive and negative valence and arousal networks. Note: Only twenty-five nodes with highest total degree are shown.

**Fig. 8:**
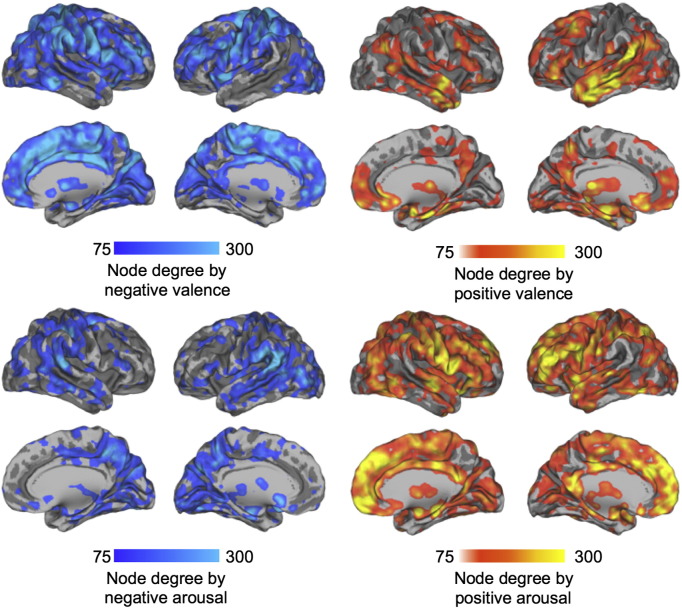
Node-degree maps highlighting how many connections from each brain voxel were statistically significantly (*p* < 0.05 FDR corrected) modulated by negative and positive valence (top) and arousal (bottom).

Valence and arousal were distinctly influencing the large-scale functional connectivity of brain areas as measured by SBPS (Fig. 5, Fig. 6, Fig. 7, Fig. 8; see electronic supplementary material for full thresholded connectivity matrices). Positive valence was associated with a small number of connectivity changes, mainly involving connections of the thalamus and amygdalae. In turn, negative valence resulted in markedly larger-scale changes in connectivity of the fronto-parietal, limbic (insula, cingulum) and fronto-opercular (primary and premotor cortices, lateral prefrontal cortex) connections, with bilateral insula and right inferior temporal cortex and bilateral superior orbital gyri being largest hubs. However, connectivity changes of the occipitotemporal cortices were not prominent.

Positive arousal was associated with widespread increase in connectivity, with prominent modulations in connections from the striatum, amygdala, thalamus, cerebellum, and dorsal frontal cortex, with frontocortical regions being the dominant hubs. Negative arousal was associated with smaller-scale connectivity changes focused strongly on middle temporal and thalamic regions, with frontocortical connectivity changes being markedly absent.

Analysis of the network properties of the aforementioned four networks confirmed that the networks are independent. Valence-positive and arousal-negative networks share some links (Jaccard similarity =0.019), as do valence-negative and arousal-positive networks (Jaccard similarity = 0.025). However, no overlap exists in the links of the valence-positive and arousal-positive, as well as valence-negative and arousal-negative networks (Jaccard similarity = 0).

### Regional effects in GLM

**Fig. 9:**
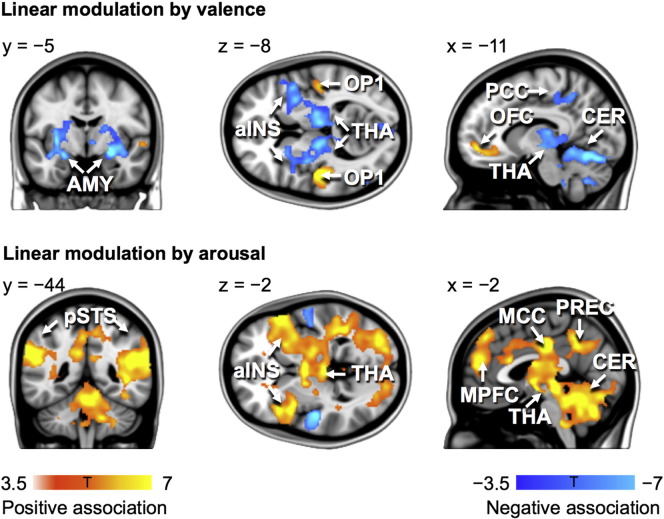
Brain regions where BOLD signal amplitude depended linearly on valence (top) and arousal (bottom) dimensions of emotional feelings elicited by the stories.

Analysis of the emotion-evoked brain responses using conventional BOLD-GLM confirmed that valence and arousal dimensions of emotions were associated with strong and spatially dissociated responses in the brain's emotion, attention and sensorimotor circuits (Fig. 4, Fig. 9). Emotional valence was positively associated with activation in orbitofrontal and primary somatosensory cortex (SI) and negatively associated with activation in limbic regions (amygdala, anterior insula, thalamus) as well as in the cerebellum. On the contrary, arousal was positively associated with activation in limbic (anterior insula, thalamus) and posterior temporal regions, (posterior superior temporal sulcus; pSTS) as well as in the midline cortical structures (medial prefrontal cortex; mPFC, anterior (ACC) and middle (MCC) cingulate cortex and precuneus) and cerebellum. Negative associations with arousal were only observed in anterior superior temporal cortices. Importantly, these effects did not in general overlap with those observed in the ISPS analysis (Fig. 4).

## Discussion

Catching emotions from spoken narratives describing real-life emotional episodes in neutral voice was associated with enhanced intersubject synchronization of listeners' brain circuitries related to emotional (ACC, amygdala, thalamus), auditory (auditory cortex), linguistic (Broca's area) and self-referential (medial prefrontal cortex) processing. The degree of synchronization across participants' brains depended linearly on the participants' current emotional state, as indexed by the arousal and valence dimensions, and the RSA analysis confirmed that having similar emotional mind states is indeed associated with having similar brain states. Finally, we demonstrate for the first time that it is possible to simultaneously track the dynamic connections between all the voxels in the brain. This allowed unravelling distinct, large-scale neural networks whose functional connectivity changed according to valence and arousal dimensions of emotions. Altogether these results, demonstrating that emotional episodes of spoken narratives make different individuals' brains ‘tick together’, provide brain-level support for the notion that emotions enhance interpersonal coherence by increasing the similarity how individuals perceive and experience their common world (Hatfield et al., 1994, Nummenmaa et al., 2012).

### Language and emotion circuits are functionally connected during emotional narrative processing

Different individuals' brain activity in the sensory projection cortices and dorsal attention network becomes synchronized during prolonged natural stimulation, such as viewing movies (Hasson et al., 2004, Jääskeläinen et al., 2008, Malinen et al., 2007) or listening to audiobooks (Wilson et al., 2008). We extend these findings by showing that similarity measures, such as ISPS (and ISC), are sensitive not only to physical stimulus features, but also to higher-order semantic and affective inferences of the sensory input (Lahnakoski et al., 2014): Even though the auditory stream remained constant in terms of low-level acoustic and prosodic properties throughout the emotional and nonemotional episodes, the ISPS fluctuated along the affective inferences the individuals drew from the semantics of the speech. Our findings thus suggest that emotional speech provides a powerful way for ‘tuning in’ different individuals: Similarity of information processing (ISPS) across listeners' specific brain circuitries was greatest during emotionally intense episodes of the narratives. This result accords with psychophysiological and social psychological studies where synchronization of emotions across individuals via spoken narratives has been demonstrated in contexts ranging from patient–therapist verbal interaction (Marci et al., 2007) to conversations at workplace (Barsade, 2002).

When participants view movies containing neutral and emotional episodes, negative valence is associated with increased ISC in the emotion-processing circuitries and in the cortical midline structures, whereas high arousal is associated with increased ISC in the visual cortices and dorsal attention systems (Nummenmaa et al., 2012). Here we observed a similar synchronization pattern using speech stimuli, with the exception that arousal was positively associated with more similar processing of speech (rather than visual information) as indicated by enhanced ISPS auditory cortices and Broca's region, thus replicating conceptually our prior results with movie stimuli (Nummenmaa et al., 2012). Arousal thus seems to have a modality-independent role in synchronizing different individuals' sensory processing of the external world. Unlike movies, our narratives did not depict sensory survival-salient events or affect-laden prosodic changes that could trigger automatic responses in the affective circuits. Instead, extracting the affective meaning from the narratives required semantic access to the narrative contents; the consequent retrieval of the emotional associations could have led to reconstruction of the corresponding sensorimotor and visceral states in the modality-specific sensory circuits of the brain (Costa et al., 2010).

Negative valence was prominently associated with stronger synchronization in midline components of the ‘default mode’ network (DMN) whose activity is typically suppressed during external stimulation or during task performance (Fox et al., 2005, Raichle et al., 2001). The valence-driven increase in ISPS in the DMN thus corroborates recent suggestions that the DMN may actually be involved in the evaluation of internal and external survival-relevant information, and also well as self-referential and social processing (Raichle et al., 2001, Schilbach et al., 2008).

Negative valence was also associated with synchronization of somatosensory cortices. This finding is in line with prior work showing that emotions trigger topographically specific patterns of bodily sensations (Nummenmaa et al., 2014a), and that the primary somatosensory cortices are activated during both emotional perception and emotional contagion (Nummenmaa et al., 2008, Nummenmaa et al., 2012) and their damage (Adolphs et al., 2000) or inactivation by transcranial magnetic stimulation (Pourtois et al., 2004) impairs recognition of others' emotions. Consequently, emotional perception could involve automatic activation of the sensorimotor correlates of the observed emotions. To further test this hypothesis, we asked an additional six participants to continuously rate how prominently bodily actions were described or implied in the stories. In line with our hypothesis, these ‘action ratings’ correlated significantly with both valence (r = − 0.30, *p* < 0.001) and arousal (r = 0.15, *p* < 0.001) ratings, suggesting a link between narrative-triggered emotions and bodily actions.

The RSA across subjects confirmed that similarity of emotional states was also associated with similarity of brain activation in sensory and supplementary motor cortices. These findings confirm that having similar mind states are literally associated with having similar brain states. Moreover, the affective synchronization of neural time courses when exposed to affective speech might be the crucial mechanism supporting mutual understanding of the world during such events: In other words, emotions seem to ‘tune’ different brains more closely to each other. It must nevertheless be stressed that our results are related to synchronous brain activation in observers exposed to similar emotional stimulation, rather than cortical synchrony between a speaker and a listener (Stephens et al., 2010). This experimental setting may explain why the synchronization effects were to some extent unspecific, extending also to the cortical midline structures. Future studies need to address how emotional semantics influence the direct brain-to-brain coupling during conversation.

### Emotions modulate large-scale functional connectivity of the brain

Prior studies have addressed emotion-triggered changes in functional brain connectivity due to discrete emotional states and with a limited set of a priori ROIs (Eryilmaz et al., 2011, Tettamanti et al., 2012). On the contrary, our SBPS analysis was able to delineate anatomically only minimally constrained large-scale brain networks underlying the processing of emotional valence and arousal dimensions, revealing that both negative and positive valence and arousal are associated with enhanced functional connectivity in large-scale networks spanning from limbic circuits to the neocortex. The present application of the dynamic, whole-brain SBPS connectivity techniques (Glerean et al., 2012) confirms that this kind of slow large-scale connectivity changes and resulting functional networks can be meaningfully analysed from the task-evoked fMRI data, and they reveal phenomena that conventional connectivity techniques cannot.

The most prominent feature of the node degree (Fig. 7, Fig. 8, Fig. 9) and connectivity (Fig. 5, Fig. 6) maps is the sheer number of connections from limbic, sensory and association cortices whose strength was modulated by valence and arousal. Emotion systems may thus manage information processing priorities not only in specific sub-systems or circuits, but also at very global level of the brain, consequently casting doubts on the feasibility of focused analysis of regional connectivity during emotional states. Two critical differences were found between the effects of valence and arousal on brain connectivity. First, arousal-modulated connectivity changes were most prominently due to increasing arousal, with markedly weaker connectivity changes due to decreasing arousal. On the contrary, both increasing (pleasant feelings) and decreasing (unpleasant feelings) emotional valence modulated the strength of a large number of connections. Second, the spatial layout was different for the connectivity changes due to valence and arousal. Whereas positive valence resulted in increased frontotemporal, thalamic and striatal connectivity, negative valence resulted in widespread increase in connections from occipito-parietal, limbic (insula, cingulum) and fronto-opercular (primary and premotor cortices, lateral prefrontal cortex) regions. On the contrary, the connectivity changes from the brain's speech processing circuit (specifically Broca's region, auditory cortex and IPC) and limbic emotion circuits (thalamus, striatum, amygdala) as well as frontal cortex were prominently associated with increasing arousal, with only a limited set of occipito-parietal connections being associated with decreasing arousal.

The number of statistically significant functional connections from the thalamus and amygdala increased significantly as a function of both high arousal as well as low and high valences. Even though the SBPS measure cannot quantify the directionality of the connections or existence of underlying anatomical connectivity pattern, it can be speculated that the thalamus and amygdala may modulate the processing of emotional information via their connections to sensory and higher-order association cortices during emotionally intensive episodes. The observed partially independent effects of valence and arousal on brain connectivity fit with the proposals that valence and arousal have distinct neurophysiological basis (Anderson et al., 2003, Posner et al., 2005) and roles in coordinating motivated behaviour (Nummenmaa et al., 2012).

### Dissociation between ISPS and task-evoked BOLD responses

The BOLD-GLM analysis revealed that distinct neural circuits tracked valence (positive: orbitofrontal cortex and SI; negative: amygdala, insula, thalamus and cerebellum) and arousal (positive: insula, thalamus, mPFC, pSTS, precuneus, cerebellum; negative: STG) dimensions of the affective significance of the narratives. This reveals that independent, yet partially overlapping brain networks track valence and arousal extracted from semantics of speech, similarly as they track the corresponding properties of biologically survival-salient sensory events, such as affective sounds (Viinikainen et al., 2012), pictures (Viinikainen et al., 2010), and odours (Anderson et al., 2003).

Our results suggest that emotion-dependent BOLD amplitude modulations and intersubject synchronization reflect different underlying neural processes: Whereas valence and arousal primarily modulated BOLD-GLM responses in the limbic and temporoparietal cortices, significant ISPS effects were observed in systems processing speech (auditory cortex, Broca's area) and self-referential information (cortical midline), where BOLD-GLM modulations were markedly absent. Thus, the emotion-driven synchronization of brain activity could reflect interpersonal coordination of higher-order representations of the internal and external world, rather than only similarity of emotion states.

At a more general level, these results accord with the proposal that analysing response similarity (or reliability) rather than amplitude may reveal phenomena that response amplitudes cannot unravel. Critically, intersubject analyses such as ISPS can take into account the entire time-dependent response profile, rather than the mere magnitude of specific response modulation. Consequently, these analyses enable more accurate characterization of the modulations of the brain activation time courses over prolonged timescales (Hasson et al., 2010). More importantly, analysing data in phase (ISPS) rather than by temporal correlations allows studying phenomena (particularly those like emotions) whose neural timing differs across individuals. All in all, these results confirm that intersubject synchronization (such as ISC and ISPS) and task-driven BOLD responses can reflect conceptually different phenomena (Nummenmaa et al., 2014b), and that they should be used in conjunction when analysing data from cognitive neuroimaging experiments.

## Conclusions

Spoken discourse provides a powerful way of inducing shared emotional states in different individuals. Catching emotions from others' speech is associated with changes in emotional feelings, activity of the autonomous nervous system and enhanced intersubject synchronization of brain activity in the emotion and speech processing circuitries. We propose that such emotional contagion provides an embodied framework that supports understanding others' mental states and may facilitate social interaction and interpersonal understanding (Hari and Kujala, 2009, Hatfield et al., 1994). This conclusion accords with the proposals that we constantly remap others' emotional states into our own corresponding sensorimotor and visceral brain areas (Jackson et al., 2005, Keysers et al., 2010, Nummenmaa et al., 2012, Preston and de Waal, 2002). Here, we demonstrate that this matching occurs not only when we see others' emotions (Nummenmaa et al., 2012), but also when we hear about them. Emotional language apparently provides a way for bringing minds together to facilitate social interaction by enhancing synchronization of thoughts, brain activations and behaviours across individuals.

The following are the supplementary data related to this article.

Fig. S-1 Average power spectra for ISPS, valence and arousal (a) and Spearman correlations between the results obtained with the frequency used in the original analysis (0.04–0.07 Hz) with those obtained using the adjacent bands as well as the slow-3, slow-4 and slow-5, separately for valence (b) and arousal (c).

Supplementary Table 1 Brain regions whose ISPS was modulated by negative and positive valences and arousal. Coordinates show locations for cluster peaks. The data are thresholded at *p* < 0.05 FDR corrected at cluster level.

Supplementary Table 2 Brain regions whose haemodynamic responses were modulated by negative and positive valences and arousal. Coordinates show locations for cluster peaks. The data are thresholded at *p* < 0.05 FDR corrected at cluster level.

Supplementary material 1 Description of the supplementary network data files.

Supplementary material 2 Arousal-negative network in Pajek NET format.

Supplementary material 3 Arousal-positive network in Pajek NET format.

Supplementary material 4 Valence-negative network in Pajek NET format.

Supplementary material 5 Valence-positive network in Pajek NET format.

## Footnotes

1 We also analyzed the data at adjacent frequency bands (shifting the band 0.01 Hz up and down); these results (Supplementary Fig. 1) show high overlap (rs > 0.7) with those reported here. Analysis of the ISPS by valence and arousal for the very slow bands (‘slow five’) shows only a positive association with arousal and sensory cortices as well as negative association with arousal and the task-negative (‘default mode’) network, and the resulting beta maps do not correlate with those obtained in the frequency band employed in our main analysis.
