# Supplementary figures and images for: Emotional speech synchronizes brains across listeners and engages large-scale dynamic brain networks
Source: Neuroimage. 2014 Nov 15;102:498–509. doi: 10.1016/j.neuroimage.2014.07.063 (PMC4229500; doi:10.1016/j.neuroimage.2014.07.063)

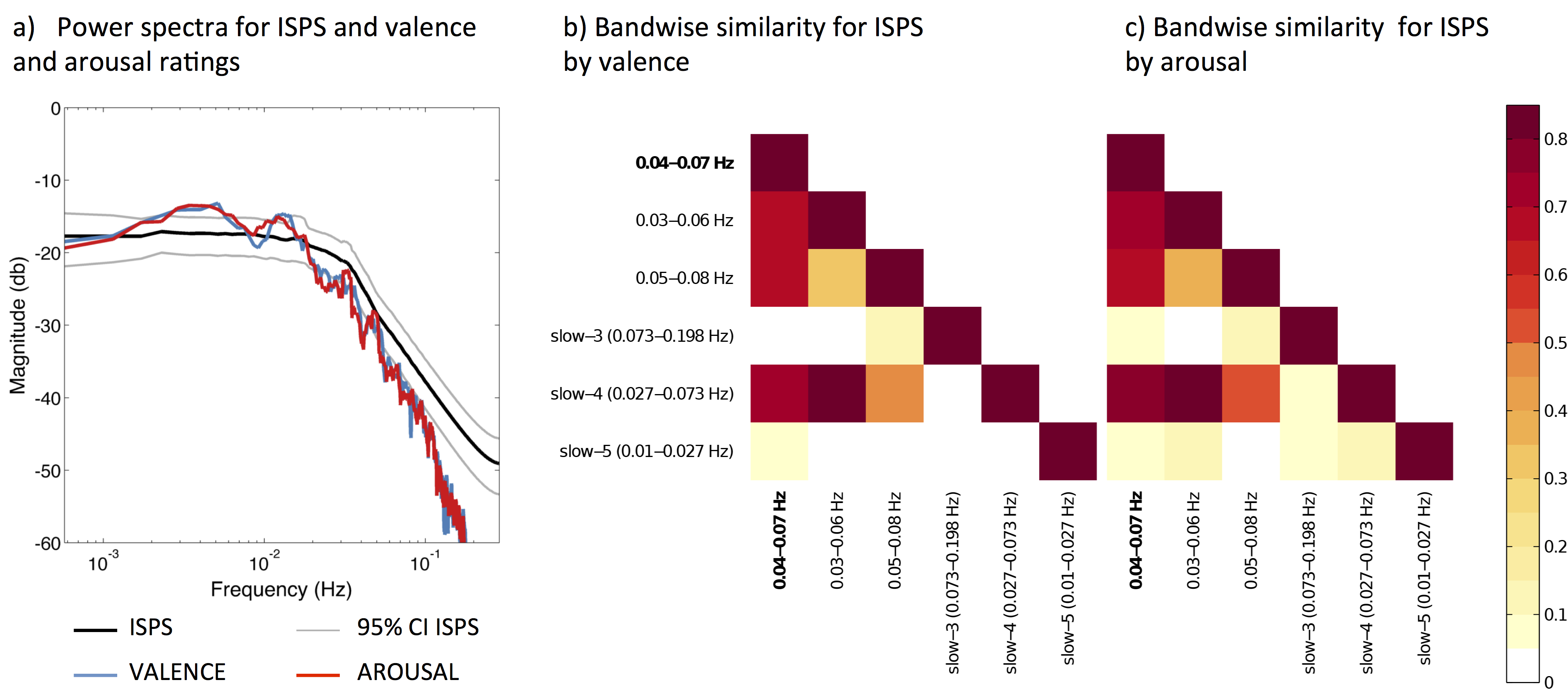

Supplement: Fig. S-1 — Average power spectra for ISPS, valence and arousal (a) and Spearman correlations between the results obtained with the frequency used in the original analysis (0.04–0.07 Hz) with those obtained using the adjacent bands as well as the slow-3, slow-4 and slow-5, separately for valence (b) and arousal (c). [file mmc1.zip › nim11565-mmc1.tiff]
