# Supplementary material for: Emotional speech synchronizes brains across listeners and engages large-scale dynamic brain networks
Source: Neuroimage. 2014 Nov 15;102:498–509. doi: 10.1016/j.neuroimage.2014.07.063 (PMC4229500; doi:10.1016/j.neuroimage.2014.07.063)
Supplement: Supplementary Table 1 — Brain regions whose ISPS was modulated by negative and positive valences and arousal. Coordinates show locations for cluster peaks. The data are thresholded at p < 0.05 FDR corrected at cluster level. [file mmc2.docx]

**Supplementary Table 1.** Brain regions whose ISPS was modulated by negative and positive valence and arousal. Coordinates show locations for cluster peaks. The data are thresholded at p < 0.05 FDR corrected at cluster level.

| **Region** | **Laterality** | **x** | **y** | **z** | **r** | **k** |
| --- | --- | --- | --- | --- | --- | --- |
| *ISPS by negative valence* |  |  |  |  |  |  |
| Anterior cingulate cortex | Left | 0 | 32 | 28 | 0.35 | 606 |
| Precuneus | Right | 16 | -56 | 30 | 0.33 | 159 |
| Lingual gyrus | Right | 18 | -66 | 4 | 0.31 | 389 |
|  |  |  |  |  |  |  |
| *ISPS by positive valence* |  |  |  |  |  |  |
| Superior occipital gyrus | Right | 30 | -78 | 46 | 0.43 | 125 |
| Middle frontal gyrus | Right | 38 | 26 | 44 | 0.39 | 649 |
| Medial frontal frontal gyrus | Right | 12 | 46 | 34 | 0.37 | 345 |
|  |  |  |  |  |  |  |
| *ISPS by negative arousal* |  |  |  |  |  |  |
| Middle frontal gyrus | Right | 26 | 28 | 28 | 0.40 | 268 |
| Middle temporal gyrus | Right | 46 | -54 | 16 | 0.37 | 327 |
| Precuneus | Left | -2 | -52 | 50 | 0.35 | 1371 |
| Lingual gyrus | Right | 10 | -64 | -4 | 0.35 | 142 |
| Middle frontal gyrus | Left | -32 | 36 | 36 | 0.32 | 196 |
|  |  |  |  |  |  |  |
| *ISPS by positive arousal* |  |  |  |  |  |  |
| Angular gyrus | Left | -40 | -64 | 36 | 0.41 | 138 |
| Cerebellum | Right | 34 | -76 | -52 | 0.40 | 133 |
| Superior frontal gyrus | Left | -8 | 44 | 32 | 0.39 | 570 |
| Middle frontal gyrus | Right | 34 | 26 | 48 | 0.39 | 142 |
| Middle orbital gyrus | Right | 44 | 52 | -14 | 0.38 | 133 |
| Superior frontal gyrus | Left | -18 | 24 | 48 | 0.35 | 260 |
| Angular gyrus | Right | 42 | -64 | 50 | 0.35 | 272 |
| Superior temporal gyrus | Right | 52 | -22 | 14 | 0.33 | 327 |
| Middle temporal gyrus | Left | -48 | -36 | 8 | 0.30 | 188 |
