# Supplementary material for: Emotional speech synchronizes brains across listeners and engages large-scale dynamic brain networks
Source: Neuroimage. 2014 Nov 15;102:498–509. doi: 10.1016/j.neuroimage.2014.07.063 (PMC4229500; doi:10.1016/j.neuroimage.2014.07.063)
Supplement: Supplementary Table 2 — Brain regions whose haemodynamic responses were modulated by negative and positive valences and arousal. Coordinates show locations for cluster peaks. The data are thresholded at p < 0.05 FDR corrected at cluster level. [file mmc3.docx]

**Supplementary Table 2.** Brain regions whose haemodynamic responses was modulated by negative and positive valence and arousal. Coordinates show locations for cluster peaks. The data are thresholded at p < 0.05 FDR corrected at cluster level.

| **Region** | **Laterality** | **x** | **y** | **z** | **T** | **k** |
| --- | --- | --- | --- | --- | --- | --- |
| *Negative valence* |  |  |  |  |  |  |
| Cerebellum | Right | 32 | -54 | -22 | 9.12 | 17197 |
| Cerebellum | Right | 8 | -40 | -46 | 6.7 | 685 |
| Precuneus | Left | -4 | -50 | 48 | 6.69 | 1666 |
| Angular gyrus | Right | 48 | -46 | 28 | 6.62 | 1709 |
| Supramarginal gyrus | Left | -54 | -48 | 28 | 6.34 | 710 |
| Middle frontal gyrus | Right | 38 | 30 | 42 | 5.88 | 320 |
| Middle occipital gyrus | Left | -42 | -72 | 18 | 4.6 | 271 |
|  |  |  |  |  |  |  |
| *Positive valence* |  |  |  |  |  |  |
| Superior temporal gyrus | Left | -46 | -18 | 6 | 9.24 | 561 |
| Orbitofrontal cortex | Left | -10 | 52 | -6 | 6.17 | 479 |
| Superior temporal gyrus | Right | 48 | -18 | 4 | 6.06 | 321 |
|  |  |  |  |  |  |  |
| *Negative arousal* |  |  |  |  |  |  |
| Superior temporal gyrus | Left | -46 | -20 | 6 | 7.45 | 1199 |
| Superior temporal gyrus | Right | 46 | -14 | 4 | 5.64 | 536 |
|  |  |  |  |  |  |  |
| *Positive arousal* |  |  |  |  |  |  |
| Supramarginal gyrus | Left | -58 | -42 | 30 | 10.7 | 53121 |
| Amygdala | Left | -38 | -4 | -16 | 5.58 | 465 |
