# Supplementary material for: Emotional speech synchronizes brains across listeners and engages large-scale dynamic brain networks
Source: Neuroimage. 2014 Nov 15;102:498–509. doi: 10.1016/j.neuroimage.2014.07.063 (PMC4229500; doi:10.1016/j.neuroimage.2014.07.063)
Supplement: Supplementary material 1 — Description of the supplementary network data files. [file mmc4.docx]

**Description of the supplementary network data files**

The supplementary network files are stored in Pajek format. They represent the significant links that were temporally co-varying with self reported valence and arousal. The suffix "_P" stands for positive relationship (e.g. for increasing valence the connectivity of the link increases) and “_N” for negative relationship.

Each node (6mm voxel) is labeled with its MNI coordinates. The networks contain only storing q<0.1 pFDR corrected values, corresponding to weights larger than ~0.2. For visualization purposes we recommend tresholding the networks with larger weights (e.g. around 0.28) to avoid cluttering.
